# An alternative method of SNP inclusion to develop a generalized polygenic risk score analysis across Alzheimer's disease cohorts

**DOI:** 10.3389/frdem.2023.1120206

**Published:** 2023-07-31

**Authors:** Keeley J. Brookes, Tamar Guetta-Baranes, Alan Thomas, Kevin Morgan

**Affiliations:** ^1^Interdisciplinary Biomedical Research Centre, Biosciences, Clifton Campus, Nottingham Trent University, Nottingham, United Kingdom; ^2^Human Genetics, Life Sciences, University Park, University of Nottingham, Nottingham, United Kingdom; ^3^Brains for Dementia Research Coordinating Centre, Institute of Neuroscience, Newcastle University, Newcastle upon Tyne, United Kingdom

**Keywords:** polygenic risk score, dementia, Alzheimer's disease, cross-cohort, predictability

## Abstract

**Introduction:**

Polygenic risk scores (PRSs) have great clinical potential for detecting late-onset diseases such as Alzheimer's disease (AD), allowing the identification of those most at risk years before the symptoms present. Although many studies use various and complicated machine learning algorithms to determine the best discriminatory values for PRSs, few studies look at the commonality of the Single Nucleotide Polymorphisms (SNPs) utilized in these models.

**Methods:**

This investigation focussed on identifying SNPs that tag blocks of linkage disequilibrium across the genome, allowing for a generalized PRS model across cohorts and genotyping panels. PRS modeling was conducted on five AD development cohorts, with the best discriminatory models exploring for a commonality of linkage disequilibrium clumps. Clumps that contributed to the discrimination of cases from controls that occurred in multiple cohorts were used to create a generalized model of PRS, which was then tested in the five development cohorts and three further AD cohorts.

**Results:**

The model developed provided a discriminability accuracy average of over 70% in multiple AD cohorts and included variants of several well-known AD risk genes.

**Discussion:**

A key element of devising a polygenic risk score that can be used in the clinical setting is one that has consistency in the SNPs that are used to calculate the score; this study demonstrates that using a model based on commonality of association findings rather than meta-analyses may prove useful.

## Introduction

The investigation of genetic predisposition to complex disease via polygenic risk scores (PRS) has increased in recent years (Plomin and von Stumm, 2022), and its popularity is guided by its long-term promise of clinical utility for early diagnosis and personalized therapeutic intervention, leading to disease prevention. Meanwhile, PRSs have the potential to further basic research by identifying causal variants, leading to novel drug development, the stratification of samples for clinical exploration, and therapeutic intervention trials involving targets who are most likely to respond.

Briefly, PRS analyses utilize large-scale genome-wide association study (GWAS) summary data (base data set) to score genotypes present in the target data set, i.e., an independent cohort of case and control samples, producing a single risk score for each individual for the disease under investigation. PRSs are calculated as a sum of the number of “effect” alleles present, weighted by their effect size (provided by the GWAS summary statistics), and are then tested for their correlation with the disease phenotype and the ability to discriminate the cases from controls using the area-under-the-curve (AUC) statistic from the receiver operating characteristic (ROC) curve. The selection of SNPs to be included in the PRS is normally determined by a significance threshold in the summary statistics, incorporating all SNPs with GWAS *p*-values below that cut-off (Purcell et al., 2009; Euesden et al., 2015).

However, this type of analysis has not been without its criticism, and the methodology is still being developed from its original and simplest form of taking significant GWAS SNPs and summing them by their weighted effect sizes (Purcell et al., 2009) to the more complex machine learning algorithms being explored (Baker and Escott-Price, 2020). A crucial caveat to the development of a clinically relevant PRS is the lack of translation of PRS models across datasets, with initial discriminability estimates failing to be maintained on additional independent cohorts (Janssens, 2019; Baker and Escott-Price, 2020). This is due to the lack of commonality of SNPs between cohorts not only because of differences in the SNP content on genotyping platforms but also potentially because of differences in allele frequencies between cases and controls in different cohorts, which governs the discriminability and is, therefore, subject to the same nuances as gene association studies (Nakaoka and Inoue, 2009; Shi et al., 2016). The lack of consistency in SNP content can be overcome by imputing data to fill in missing genotypes between cohorts or increase the coverage of SNPs between the target and base data sets. However, there are concerns about imputation accuracy and its effect on the subsequent analysis for SNPs with low minor allele frequencies, low levels of linkage disequilibrium, and those that may have gone through recent and dramatic allele frequency changes (Aleknonyte-Resch et al., 2021; Ali et al., 2022). The International Genomics of Alzheimer's Project (IGAP) Stage 1 (IGAP_S1) GWAS summary statistics (Lambert et al., 2013) are often used as a base data set for PRS studies in Alzheimer's disease (AD), consisting of more than 7 million imputed genotypes. During the imputation and phasing of genetic data, some genotypes are lost in the process, for example, the rs9271192 (*HLA-DRB5*) SNP, which was one of the GWAS hits from the Lambert et al. (2013) study but was absent from the summary statistics, presumably having been lost in the imputation process. Furthermore, the focus in PRS studies is often on the ability to accurately identify cases from controls, with the extent of SNP commonality between the models developed across studies still to be fully explored to identify a consistent set of SNPs that could be used for clinical purposes.

Further commentary suggests that the traditional pruning/clumping of SNPs, selecting those that are the most significant (denoted as the “Index SNP”) within blocks of linkage disequilibrium (LD), followed by the “thresholding” for SNP inclusivity, falls short of explaining the heritability estimates and could discard information that increases prediction accuracy (Vilhjálmsson et al., 2015). Conversely, relaxing the thresholds to include SNPs outside the GWAS significance threshold has yielded the PRS models utilizing thousands of SNPs, yet whilst this has provided greater discriminability (Escott-Price et al., 2015), the clinical utility of including such SNPs, often with negligible effect sizes, is questionable; if they do not impact the biology of the disease or suggest a druggable target, are they useful to include (Janssens, 2019).

This investigation utilized the summary statistics from the Lambert et al. (2013) study and a cross-cohort methodology for SNP selection for PRS model generation in AD (see the Data Availability section for details of the cohorts used). The incorporation of SNPs into the PRS model is traditionally determined by their significance in the summary statistics base data set; however, the discrimination of cases and controls rests with the allele frequency difference of the selected SNPs in the target data set. Incorporating SNPs with large allele frequency differences between the cases and controls in the target data set will lead to more divergent PRSs between these two groups and, consequently, a more accurate prediction model. In contrast to the traditional thresholding method, in this study, SNPs were selected based on their consistent contribution to a highly discriminatory PRS model across several cohorts. It was hypothesized that, by doing this, SNPs that underlie the disease will be selected and improve the translatability of the PRS model across different data sets. The developed PRS models were then validated with three independent AD cohorts, showing an average of more than 70% accuracy in discriminability across all cohorts.

## Methods

### Data sets

#### Base data set

Summary statistics were obtained from the Lambert et al. (2013) study. These summary statistics were generated from imputation during the first stage of this study (IGAP_S1) by conducting a meta-analysis of four GWAS samples of European ancestry (*n* = 17,008 cases, *n* = 37,154 controls). This data set was selected because of the number of SNPs in the summary statistics and because of generating the number of samples that had a diagnosis of AD.

#### PRS development data sets

Five genotyping data sets were utilized for this study; see the Data Availability section for details on the cohorts. The data sets used were the Brains for Dementia Research (BDR) cohort, as described in a previous study by this group (Young et al., 2021), and four cohorts previously utilized in large GWAS studies, which are freely available for download: ADC7, NIA, ROSMAP and TGEN data sets. ROSMAP data were obtained from the AMP-AD Knowledge Portal via the Synapse Data Access System (https://www.synapse.org/). The ADC7, NIA, and TGEN data sets were downloaded from NIAGADS (https://www.niagads.org/). The data sets that were selected had been genotyped on different platforms, had varying sample sizes, and had *APOE* isoform/genotype data (Supplementary material 5).

All data sets underwent quality control with PLINK v1.9 (Purcell et al., 2007), and SNPs with a minor allele frequency of <1% were removed. In addition, genotype calls of <95% and those deviating significantly from Hardy–Weinberg equilibrium (*p* < 0.0001) in the control samples were also removed. Furthermore, from the available information, non-Caucasian samples including Hispanic samples from the ROSMAP data set were also removed. Only samples with a definite or confirmed diagnosis of AD were included. When the genotyping of *APOE* isoform SNPs (rs429358 & rs7412) was not included in the genotype data, the isoform information in the accompanying clinical data files for each data set was used to determine the likely SNP genotypes.

#### Validation data sets

Three validation data sets (MTC, WashU, and TARCC) were also obtained from NIAGADS (https://www.niagads.org/), and details on these cohorts can be found in the Data Availability section. Samples from these data sets were included in this study if diagnosed as AD or control and had *APOE* genotyping. Details of the sample sizes and genotyping platforms used to generate their data are available in Supplementary material 5.

### Clumping and LD clump SNP assignment

The IGAP_S1 summary statistics were clumped using the 1000Genomes European data set in PLINK v1.9 (Purcell et al., 2007), with the parameters –clump-p1 1, –clump-p2 1, –clump-kb 250, and –clump-r2 0.8. These parameters were then used to define the LD clumps across the base data set SNPs. The IGAP_S1 clumped output file consists of rows of clumped data, with the most significant SNP of the clump denoted as the “Index” and all other SNPs that reside within that clump in subsequent columns. Traditionally, only these Index SNPs that are in common with the target dataset are used in PRS modeling.

In this investigation, the following alternative steps were taken to identify SNPs to be included in the PRS modeling (the R Script is available on request):

1. The clump/LD blocks identified in the IGAP_S1 data were numbered, and each SNP from the IGAP_S1 data that resides in that clump was “tagged” with its corresponding clump/LD number.
2. Clump Tag SNPs in the IGAP_S1 summary statistics were matched by SNP ID with those in each development data set.
3. A single SNP representing each clump was selected for each development data set.
4. Clumps that had representative SNPs across all five of the development data sets were taken forward to create the “Common Clump SNP set” for analysis.
5. The beta effect size scores and reference alleles were obtained from the IGAP_S1 summary statistics for all SNPs identified for the polygenic risk score algorithm, producing five sets of SNPs to be used, one for each of the development cohorts.

### Common clump SNP set quality control

SNPs representing each clump across the PRS development data sets were investigated for large deviations in beta effect sizes (obtained from the IGAP_S1 and used to generate the risk scores in the target data sets). The SNPs tagged in each clump across the data sets were examined for differences in beta effect direction and those that displayed a standard deviation (*SD*) of ±1 across the beta effect size scores. Clumps with opposing directions of beta effects or *SD* of > 0.5 were removed; this resulted in the removal of 1,067 clumps based on the differential direction of the beta values only. No further clumps were removed based on the beta value *SD* of the five SNPs representing the clump across the five PRS development data sets; the maximum *SD* observed was 0.003. In addition, a total of 42,684 clump-tagged SNPs were available for analysis per cohort.

### Threshold modeling

SNPs from the base data set at each IGAP_S1 significance threshold from 5 × 10^−8^ to 1, increasing at intervals of 10^−6^, were used to generate the score file for the PRS modeling using the –score parameter in PLINK v1.9 (Purcell et al., 2007). Logistic regression was carried out in R v4.0.3 (R Core team, 2021), followed by calculating the AUC (accuracy of the model to discrimination case from control) using the pROC package (Robin et al., 2011). The results are presented in Supplementary material 5. R The scripts are available upon request.

### Perfect discrimination modeling

SNPs were recruited into the PRS model on an individual basis based on the *p-*value of association (generated in PLINK –assoc analysis) in the developmental data set and were used to generate the score file for PRS modeling using the –score parameter in PLINK v1.9 (Purcell et al., 2007). Logistic regression was carried out in R v4.0.3 (R Core team, 2021), followed by calculating the AUC using the pROC package (Robin et al., 2011). The results were used to determine the effect that the addition of each SNP had on the AUC (either increase or decrease) and labeled it with the effect direction. The SNPs were then re-ordered by *p-*value and the increase in AUC effect before undergoing single SNP addition PRS modeling to generate perfect discrimination curves, that is, AUC = 1, where all samples above a certain risk score were cases and all the samples that were below were controls.

### Generation of absolutes

“Absolute” controls and cases were artificially generated to represent the absolute minimum score (control) and the absolute maximum score (case) that could be achieved for each SNP model. This involved creating genotypes for individuals that were homozygous for all the SNPs with effect allele beta scores with a protective (minus) direction, and an individual with the opposing genotypes. These “absolutes” were then subjected to risk scoring using the generalized PRS model to create the minimum and maximum scores that could possibly be achieved, which were then used to calculate centile bins (100 bins) at equal intervals across the entire range of possible scores. The application of risk scoring helped determine the visualization of the true spread of possible scores and where the scores obtained from the data sets resided on this full scale.

## Results

Clumping was carried out on the IGAP_S1 summary statistics with an *r*^2^ of ≥ .8 in PLINK v1.9 (Purcell et al., 2007). The coverage of the genome was explored using the BDR cohort using the traditional method of utilizing only the “Index” SNP and then any SNP in the LD clump. The greater coverage of the LD clumps of the IGAP_S1 data set was achieved by allowing any SNP that was representative of an LD block into the PRS analysis rather than just the Index SNP (Supplementary material 1). Previous A previous study (Farrell and Brookes, 2022) suggests that additional SNPs within the surrounding region of *APOE* isoform SNPs (rs429358 and rs7412) could possibly be independently contributing to the AD phenotype, and therefore, the entire *APOE* region was retained in this analysis.

In addition to the BDR data set, four additional AD genotyping data sets (ADC7, NIA, ROSMAP, and TGEN) obtained from the NIAGADS data repository were selected across a range of genotyping platforms to develop the PRS model (Supplementary Table 5). Each SNP from the cohort was “tagged” with a clump number, and clumps that were covered by SNPs in all five of the PRS developmental data sets were included in the analysis (44,291 clumps). Cross-cohort SNPs for each clump were explored for consistency in the reference allele, the direction of effect, and the beta coefficient values. When the direction of effect and the reference allele differed between the SNPs in the same LD clump, the clump was removed. The beta coefficient values were found to be very similar, with an average *SD* of 0.003 and a maximum observed *SD* of 0.1 observed across the cohorts for each LD clump. A total of 42,684 LD clumps uniquely tagged by cohort-relevant SNPs were available for PRS model generation as opposed to 33,679 SNPs in common across the cohorts, increasing the coverage of the genome and commonality between data sets.

Traditional PRS modeling was applied to these data sets for comparison, since this methodology mimics that of the PRS software tool PRSice (Euesden et al., 2015), where SNPs are recruited into the model based on the *p-*value threshold in the base data set from 5 × 10^−5^ to 1 at an interval of 10^−6^. These SNP models were subjected to logistic regression, and an AUC was generated from the R software package pROC. The best PRS models were identified for each PRS development cohort in terms of the most significant logistic regression *p-*value and AUC (Supplementary material 2, **Panel A**, and Supplementary material 5). The results were highly variable between cohorts, with the best models utilizing between 18 and 41,216 SNPs and achieving an average discrimination accuracy of 0.76 (*SD* 0.138).

Perfect discrimination models were applied to the 42,684 LD clumps of each cohort. Briefly, SNPs tagged in each clump were ordered by cohort significance values and incorporated into the PRS model sequentially (Supplementary material 2, **Panel B**). These SNPs were then reordered based on their cohort significance and positive direction of effect on the AUC (Supplementary material 2, **Panel C**). All SNPs contributing to the point where perfect discrimination (AUC = 1) was first achieved were taken from each cohort. Two thresholds of commonality were set for testing: a lenient threshold requiring the LD clump to contribute to perfect discrimination in two of the five cohorts and a stringent threshold requiring the LD clump to contribute to the perfect discrimination in three of the five cohorts.

The lenient threshold identified a 2207-LD-clump PRS model, which, when applied to the development cohorts, yielded highly significant correlations and discriminatory accuracies of over 90% in four of the cohorts. The ROSMAP cohort demonstrated an accuracy of 53.82%, with the PRSs showing no significant correlation with disease outcome (Table 1). When they were applied to the lower discriminability estimates of the validation cohorts, although each of these cohorts was missing several of the LD clumps that contributed to the model, only a small number of alternative SNPs tagging the LD clumps were identified (Table 1).

**Table 1:** Results of the lenient PRS model, in the development cohorts and validation cohorts, indicating a high discriminability of the model in the development cohorts but a significant drop in the validation cohorts.

**Development**	**SNP platform**	**# SNPs from PD contributing to PRS model**	**Logistic regression *P*-value**	**Area under the curve**
ADC7 *N =* 1462	HumanOmni Express	1761	4.32 x 10^−85^	0.9004
BDR *N =* 520	NeuroChip	612	1.12 x 10^−30^	0.9259
NIA *N =* 1,386	Human 610-quad	932	1.89 x 10^−76^	0.9463
ROSMAP *N =* 240	Affymetrix6.0/IlluminaOmni Quad	41	0.397	0.5382
TGEN *N =* 1,510	Affymetrix 6.0	1,221	7.07 x 10^−84^	0.9357
**Validation**	**SNP Platform**	**# SNPs available**	**Logistic regression** ***P*****-value**	**Area under the curve**
MTC *N =* 356	HumanOmni Express	ADC7 Panel 2204 SNPs (7 alt)	2.75 x 10^−8^	0.6948
WashU *N =* 131	HumanOmni Express	ADC7 Panel 1862 SNPs (22 alt)	0.064	0.6288
TARCC *N =* 491	Affymetrix 6.0	TGEN Panel 2070 SNPs (74 alt)	8.17 x 10^−14^	0.7062

The lenient model consists of SNPs tagging the 2207 LD clumps identified to be contributing to the perfect discrimination (PD) model in at least 2 of the development cohorts. In the top panel, the results from the development cohorts indicate the number of LD clumps from that cohort that were found in at least one other cohort, note that there is no correlation between the number of clumps identified in each cohort and the accuracy in the model. In the bottom panel, the results from the validation cohorts indicate the SNP panels used in the PRS and the number of alternative (alt) SNPs that had to be found to substitute for SNPs missing in that cohort.

Using the stringent threshold, 149 LD clumps were identified as contributing to at least three of the perfect discrimination models, and although significant correlations were achieved, the discriminability of this model was only ~70% accurate in the development cohorts, with ROSMAP again showing less accuracy than the other data sets (Table 2). Even though some of the LD clumps were missing, the validation cohorts demonstrated similar accuracies in discrimination as the development cohorts.

**Table 2:** Results of the stringent PRS model in the development cohorts and validation cohorts, indicating a more uniform discriminability of the model consisting of SNPs tagging the 149 LD clumps identified to be contributing to the perfect discrimination (PD) model in 3 out of the 5 development cohorts.

**Development**	**SNP platform**	**# LD clumps contributing to PD model**	**Logistic regression *P*-value**	**Area under the curve**
ADC7 *N =* 1462	HumanOmni express	137	1.19 x 10^−44^	0.7273
BDR *N =* 520	NeuroChip	83	6.89 x 10^−22^	0.7911
NIA *N =* 1386	Human610-quad	107	4.65 x 10^−72^	0.8259
ROSMAP *N =* 240	Affymetrix6.0/IlluminaOmni Quad	8	0.449	0.5477
TGEN *N =* 1510	Affymetrix 6.0	116	1.43 x 10^−54^	0.7734
**Validation**	**SNP platform**	**# SNPs available**	**Logistic regression** ***P*****-value**	**Area under the curve**
MTC *N =* 356	HumanOmni Express	ADC7 Panel 149 SNPs (1 alt)	1.28 x 10^−11^	0.7294
WashU *N =* 131	HumanOmni Express	ADC7 Panel 127 SNPs (2 alt)	0.035	0.6150
TARCC *N =* 491	Affymetrix 6.0	TGEN Panel 143 SNPs (6 alt)	8.32 x 10^−13^	0.7006

In Panel A, the results from the development cohorts, indicate the number of LD clumps from that cohort that were found in at least 2 other cohorts, note that there is no correlation between the number of clumps identified in each cohort and the accuracy in the model. In Panel B, the results from the validation cohorts indicate the SNP panels used in the PRS and the number of alternative (alt) SNPs that had to be found to substitute for SNPs missing in that cohort.

**Figure 1:**
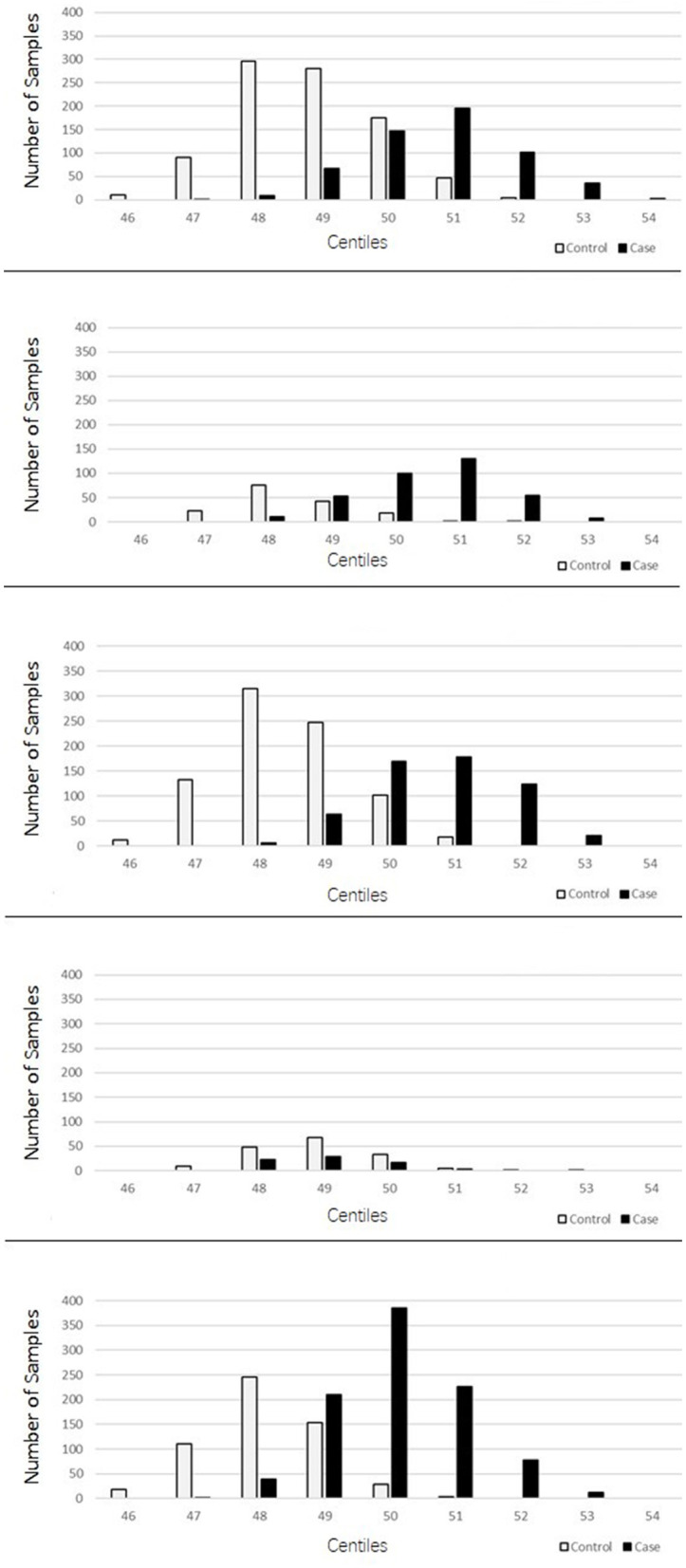
Bar chart for each cohort showing the numbers of cases (black) and controls (light gray) in each centile of the 2207 lenient model showing two-distinct normal distribution curves for the PRS corresponding to the controls and cases for each dataset.

**Figure 2:**
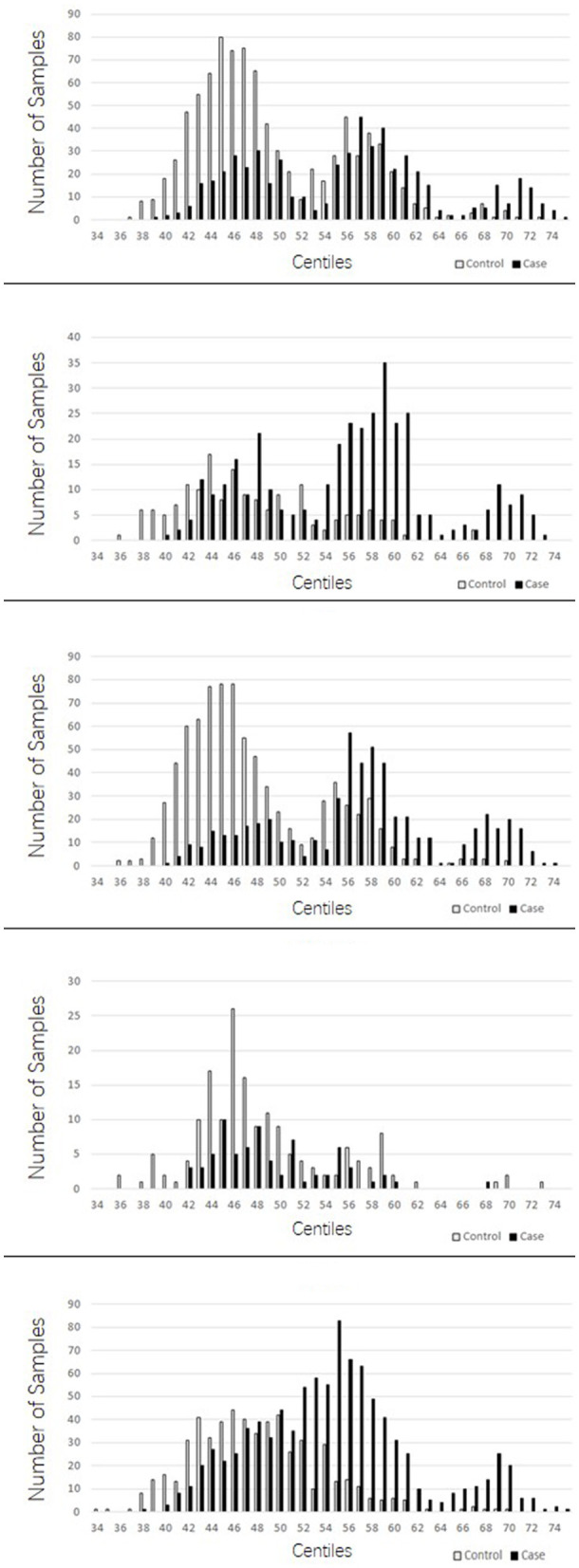
Bar chart for each cohort showing the numbers of cases (black) and controls (light gray) in each centile of the 149 stringent model. In contrast to the lenient model with more LD blocks utilized, the 149 suggests three distinct normal distributions of scores; one at the lower end of the scale made up predominantly of controls, a middle range distribution of both cases and controls, and a high range peak made up predominantly of cases.

The PRSs for “absolute” controls and cases were calculated and used to create a centile plot of scores within each data set for each model (Figures 1, 2).

The lenient model of 2,207 SNPs demonstrated a narrow range of scores that fall within the 46th to 54th centiles. Furthermore, there was an overlap of scores for cases and controls in the middle ranges; however, in all data sets, we observed two distinct peaks of scores, one for controls (48th−49th centile) and one for cases (50th−51st centile), with a 2- to 3-percentile difference between them. The ROSMAP data set has the smallest sample size and had insufficient cases in the higher score ranges, leading to a more predictive accuracy value. The stringent model of 149 SNPs created a far more dynamic range of scores from the 34th to the 75th centile; with this model, an interesting pattern of three distinct normal distributions was observed: one in the lower range from approximately the 38th to the 50th percentile, which consisted of a higher number of control samples; one in the higher range from the 64th to the 74th centile, consisting almost entirely of AD cases; and a middle third peak ranging from the 52nd to the 62nd centile, where there was an overlap of controls and cases but generally was biased toward AD case individuals. This model also indicated that the ROSMAP sample lacks AD cases with risk scores in the higher ranges, accounting for the lack of discriminability.

The stringent model had the best coverage of LD blocks in the replication samples, and therefore, the predictability of AD cases being present in the highest ranges was explored. This range of centiles suggests that more than 90% of individuals with scores 0.006 (63rd centile) or above will have AD, and this was supported in two out of the three replications cohorts. From the MTC cohort, 96.6% of individuals who scored above this threshold were AD cases. Similarly, in the TARCC cohort, 89.6% were AD cases. The WashU cohort did not support this fact, for the majority of the samples were controls for this score range; however, note that the number of individuals in this range was approximately a third of those in the other two replication cohorts.

A full table of the 2,207- and 149-model SNPs/LD blocks and genes located in the vicinity can be found in Supplementary material 3, 4. Supplementary material 5 shows a summary table of the cohorts used and the PRS models created in this study, in addition to the average AUC obtained across the five data sets and when the ROSMAP data set was removed from the mean.

## Discussion

This investigation explored an alternative method for developing a PRS model to predict AD, aiming to improve the translatability of the model to other cohorts and identify areas of the genome that may house actionable targets for therapeutic intervention. Instead of modeling the data on single SNPs that are common between the base and target data sets, it used SNPs that tag linkage disequilibrium blocks that are common between different cohorts to improve the coverage of the genome and transferability to other cohorts. As an alternative to modeling the SNP inclusion based on significance value in the base data set, SNP inclusion was based on over-fitting the data to create perfect discriminatory models for five cohorts and then identifying the linkage disequilibrium blocks that repeatedly contribute to the AUC = 1 across the development cohorts. Lenient and stringent models were applied to both the cohorts they were developed on and three additional independent cohorts to test replicability. In all cases, the average AUC achieved in the validation cohorts was lower than that in the development cohorts; however, the stringent model demonstrated an average decrease in AUC of 0.05 compared to an average decrease in AUC of 0.17 in the lenient model.

One of the main caveats for PRS analysis is the generalization of the models developed to additional cohorts. Some authors suggest that even more GWAS data than those used in this study are required to accurately calculate the effect sizes of risk alleles; conversely, the current GWAS results could be inflated, subject to the “winner's curse” (Shi et al., 2016) and the admixture of samples from the pooling of multiple cohorts (Janssens, 2019).

Preliminary unpublished explorations of AUC results on a single target dataset using a GWAS SNP model and effect sizes from different GWAS summary statistics have demonstrated that differences in the effect sizes of reference alleles do not alter the accuracy of discriminating cases from controls. Furthermore, the pattern of the AUCs obtained during the threshold modeling process in this study (Supplementary material 2) would also support the fact that, when effect sizes and SNP inclusion are similar, different AUCs can be obtained, suggesting that it is the allele frequency of the SNP in the target data set that determines the discriminatory accuracy, which is similar to the nuisances of gene association studies and significance. Therefore, an alternative hypothesis would be to identify SNPs that consistently display allele frequency differences that contribute to a more diverse risk score between cases and controls for a generalized AD risk model.

This alternative method of incorporating SNPs individually into the model based on their associated *p-*value within the target data set and directional effect on the AUC provided a model that perfectly separated the cases and controls. Importantly, when this was applied across multiple data sets, the common SNPs/LD clumps that appeared in at least two datasets (lenient model) provided a model that offered better and consistent discriminability across data sets (mean AUC = 0.8493, *SD* = 0.17) compared to when the traditional method of selecting SNPs on significance threshold models was used (mean AUC = 0.7621, *SD* = 0.14). Applying the stringent model resulted in an average AUC in the development cohort that was slightly less (mean AUC = 0.7331, *SD* = 0.11) than that of the traditional method; however, while the traditional method utilized vastly different SNPs in each model, the stringent model utilized the same SNPs in each cohort.

The ROSMAP data set was the exception, performing not as well as the other development cohorts in both models (Supplementary material 5). The ROSMAP data set sample size was the smallest of the development cohorts due to selecting only samples that had a confirmed diagnosis of AD and removing those classified as possible/probable cases of AD. Importantly, the *APOE* isoform SNPs were not significantly associated with the AD phenotype in this cohort (*p* > .05) and did not demonstrate discriminability in the PRS consisting of only these two SNPs (Supplementary material 5). As *APOE* is such a strong predictor for an AD diagnosis, the absence of this effect may go some way in explaining the lack of discriminability for this PRS compared to the other cohorts when the rs429358 and rs7412 SNPs are included in the model.

As shown in Supplementary material 3, 4, the genes located in the vicinity of the SNPs/LD blocks used in the models are those familiar in AD genetics, including not only established GWAS-hits genes, such as *APOE, TOMM40, CLU, EPHA-AS1, PICALM, CD2AP, SLC24A4*, and *MSA4A6A*, but also lesser known gene associations from previous studies and those that overlap with linkage peaks from early genetics investigations (Brookes and Morgan, 2017), such as *PACRG* (Sirkis et al., 2016); *LRAT* (Abraham et al., 2008), *UNC5C* (Jiao et al., 2014; Wetzel-Smith et al., 2014); *AKAP6* (Seshadri et al., 2010); *DLG2* (Lawingco et al., 2020; Prokopenko et al., 2022); and *DAB1* and *ARID1B* (Harold et al., 2009). It is also promising that some novel genes (*UMAD1, ABCAC1, SNX1, APP*) found in a recent GWAS study with a very large sample size were also identified in the 2207 model (Bellenguez et al., 2022).

A large proportion of the SNPs in the model produced here (81% and 74% for lenient and stringent models, respectively) had IGAP_S1 *p-*values of above .05 and, therefore, may have been omitted in traditional thresholding analysis. Furthermore, the beta coefficient values of these SNPs are not minuscule (>0.0019 and < -0.0012), suggesting that they might have observable biological effects and could be therapeutic targets.

Being that the IGAP_S1 summary statistics used in this study were already imputed, the further imputation of the genetic data of the cohorts in this study would not be beneficial due to increased variability that was observed in imputation in other data sets (Chen et al., 2020). As an alternative measure, this investigation employed clumping the base data set and selected SNPs within the target data set to capture these LD blocks, taking the Index (most significant SNP in the clumped base data set) when possible or an alternative SNP in the same clump if the Index SNP was not present. This allowed a greater coverage of the genome both within our initial analyses of the BDR cohort and the cross-cohort analyses without imputation. Interestingly, even though the validation cohorts were selected for genotyping panels to match those in the developmental cohorts, not all SNPs/LD clumps were present for analysis, possibly due to those SNPs failing to genotype in that cohort or perhaps being removed at quality control.

The additional target data sets utilized are a key limitation of this study as they are not independent from the base data sets. The IGAP study was a milestone collaborative investigation that agglomerated multiple AD cohorts from around the world into a single meta-analysis to elucidate our currently well-established AD candidate genes (Lambert et al., 2013); however, this leaves few data sets that are completely independent of this work. A previous study (Escott-Price et al., 2017) also utilized a sample cohort that was part of the IGAP study. In their analysis, they recalculated the prediction accuracy based on the SNPs from the independent data set and found that it produced a similar AUC. Therefore, given the relatively small sample size of each of the cohorts utilized in this study, it is likely that the AUCs presented are not greatly biased.

One caveat for the LD clump approach is that, due to some alleles having effect sizes in the opposite direction, 1,067 clumps were removed from the analysis as their inclusion may have produced variation at the individual level, altering the risk “centile” in which the individual may reside. The scoring algorithm for PRS normally concerns the beta value of only the reference/effect allele; however, to allow more parity, perhaps the effect sizes of both alleles could be incorporated into this calculation, especially as the opposing allele may not have a neutral effect as assumed in the current scoring algorithm in PLINK.

Compared to other PRS investigations in AD, this investigation opted for a higher *r*^2^ value of 0.8 for clumping and included genetic variation surrounding the *APOE* gene. It is possible that the additional SNPs are tagging the effects of the *APOE* isoform SNPs, leading to inflated AUC values; however, what has been presented in this article is consistent with previous studies (Harrison et al., 2020; Leonenko et al., 2021). Regarding the *APOE* region, the LD between SNPs is generally low, with only moderate (*r*^2^ < 0.56) LD observed between SNPs in this region and the rs429358 and rs7412 isoform SNPs (Young et al., 2021). Whilst the independent association with and contribution of several SNPs to AD within this area are supported by multiple studies (Huentelman et al., 2010; Rao et al., 2018; Zhou et al., 2019), the extent to which the isoform SNPs are influencing these observations is still an area for investigation.

Furthermore, this study did not include environmental predictors for AD. The inclusion of “non-genetic” variables into PRS analyses is considered by some to be problematic, as they may not be entirely independent of genetic influences already being entered into the model. Quantitative genetics studies suggest that, due to genetic–environment correlations, ~25% of environmental measures are heritable (Kendler and Baker, 2007). Female sex is viewed as a risk factor for AD, and this is genetically controlled. Therefore, to incorporate being female as a risk factor into the analysis may have increased the prediction accuracy. Similarly, longevity has also been found to have a genetic component (Herskind et al., 1996), and thus, age at death was also omitted from the model.

In conclusion, this study aimed to provide an alternative to current PRS methods for developing a model that can be applied across multiple cohorts genotyped on various platforms using informative SNPs common across data sets. This is still a hypothetical model and requires further exploration, refinement, and testing on additional data. The authors invite other fellow researchers to test this model in their own cohorts, as additional data sets will help identify additional common SNPs and refine the model by removing false positives. When SNP or LD block consistency is achieved across studies, the biological consequences may then be identified and lead to the fulfillment of the clinical utility the PRS aims to bring.

## Data availability statement

The Religious Orders Study and Memory and Aging Project, **ROSMAP**, Study genotype data were obtained from the AMP-AD Knowledge Portal via the Synapse Data Access System (SYN3219045, https://www.synapse.org/), with details about this cohort found in De Jager, P., Ma, Y., McCabe, C. et al. A multi-omic atlas of the human frontal cortex for aging and Alzheimer's disease research. Sci Data 5, 180142 (2018). https://doi.org/10.1038/sdata.2018.142. The genotypes for the Brains for Dementia Research **BDR** Cohort datasets are freely available via the Dementia Platform UK (https://www.dementiasplatform.uk/), with details about this cohort described in Young J, Gallagher E, Koska K, Guetta-Baranes T, Morgan K, Thomas A, Brookes KJ. Genome-wide association findings from the brains for dementia research cohort. Neurobiol Aging. 2021 Nov; 107:159–167. 10.1016/j.neurobiolaging.2021.05.014. The Alzheimer's Disease Centres seventh set of ADC genotyped subjects used by the Alzheimer's Disease Genetics Consortium (ADGC) **ADC7** (NG00071), the National Institute on Aging Genetics Initiative for Late-Onset Alzheimer's Disease (NIA-LOAD), **NIA** (NG00020), **TGen II**, TGEN (NG00028), samples collected by the Knight ADRC at Washington University, **WashU** (NG00097), The Texas Alzheimer's Research Care Consortium, **TARCC** (NG00097) and University of Miami/ Texas Alzheimer's Research Care Consortium Wave 2/Case Western Reserve University, **MTC** (NG00096) datasets were downloaded from NIAGADS (https://www.niagads.org/). Details of the ADC7 and WashU cohort were first published in Kunkle, B.W., Grenier-Boley, B., Sims, R. et al. Genetic meta-analysis of diagnosed Alzheimer's disease identifies new risk loci and implicates Aβ, tau, immunity and lipid processing. Nat Genet 51, 414–430 (2019). https://doi.org/10.1038/s41588-019-0358-2.

## Ethics statement

The Brains for Dementia Research cohort use for genetic analysis was reviewed and approved by the London-City and East NRES committee 08/H0704/128+5. The patients/participants provided their written informed consent to participate in this study.

## Author contributions

KB: study conception and design, data analysis and interpretation, manuscript preparation, and funding acquisition. TG-B: laboratory work. AT: provision of resources. KM: funding acquisition and manuscript review and editing. All authors contributed to the article and approved the submitted version.
